# Bioaccessibility of Antioxidants in Blackcurrant Juice after Treatment Using Supercritical Carbon Dioxide

**DOI:** 10.3390/molecules27031036

**Published:** 2022-02-03

**Authors:** Urszula Trych, Magdalena Buniowska, Sylwia Skąpska, Ireneusz Kapusta, Krystian Marszałek

**Affiliations:** 1 Department of Fruit and Vegetable Product Technology, Prof. Waclaw Dabrowski Institute of Agricultural and Food Biotechnology—State Research Institute, 36 Rakowiecka St., 02532 Warsaw, Poland; sylwia.skapska@ibprs.pl (S.S.); krystian.marszalek@ibprs.pl (K.M.); 2 Department of Dairy Technology, Institute of Food Technology and Nutrition, University of Rzeszow, Ćwiklinskiej 2D St., 35601 Rzeszow, Poland; mbuniowska@ur.edu.pl; 3 Department of Food Technology and Human Nutrition, Institute of Food Technology and Nutrition, University of Rzeszow, 2D Zelwerowicza St., 35601 Rzeszow, Poland; ikapusta@ur.edu.pl

**Keywords:** bioaccessibility, supercritical carbon dioxide, blackcurrant, vitamin C, anthocyanins, antioxidant activity, in vitro digestion model

## Abstract

Blackcurrant juice (*Ribes nigrum* L.) was subjected to supercritical carbon dioxide (SCCD) at 10, 30, and 60 MPa for 10 min at 45 °C, as well as thermally treated at 45 and 85 °C for 10 min to determine the stability, antioxidant capacity (AC), and bioaccessibility (BAc) of vitamin C, total anthocyanins, and their individual monomers. An in vitro gastrointestinal digestion model completed with dialysis was used to assess BAc. The use of SCCD at each of the pressures applied improved the stability of vitamin C, total anthocyanins, and AC before in vitro digestion. As a result of digestion, the total content of vitamin C, anthocyanins, and AC decreased. The highest BAc of vitamin C was noted in fresh juice (FJ) (40%) and after mild heat treatment at 45 °C (T45) (46%). The highest BAc of total anthocyanins was also noted in the FJ (4.4%). The positive effect of the application of SCCD on the BAc of the delphinidin-3*-O-*glycosides was observed compared to T45 and thermal pasteurization at 85 °C (T85). Although SCCD did not significantly improve the BAc of vitamin C and total anthocyanins, the higher AC of SCCD samples after intestinal digestion (ABTS+• and DPPH•) and in dialysate (ABTS+•) compared to thermally treated was observed. The protocatechuic acid was detected by UPLC-PDA-MS/MS as the major metabolite formed during the digestion of delphinidin-3-*O*-rutinoside. This may indicate the influence of SCCD on improvement of the accessibility of antioxidants for digestion, thanks to which more metabolites with high antioxidant activity were released.

## 1. Introduction

Growing awareness of the impact of proper nutrition on the health and quality of human life is one of the most important topics for food technologists, especially with regard to the application of emerging techniques for food preservation. Modern food preservation techniques, such as treatment with supercritical carbon dioxide (SCCD) might be a good alternative to thermal heating since it allows maintaining a high antioxidant content while ensuring food safety and high sensorial properties [1,2,3]. Supercritical CO_2_ is a non-toxic, inexpensive, readily available, non-flammable medium that is approved as safe. It is characterized by a low viscosity (3–7 × 10^−5^ Pa s) and zero surface tension, thanks to which it is able to quickly and effectively penetrate the sample [4]. SCCD carried out at a pressure ranging from 7.28 up to 60 MPa allowed for the effective reduction of microorganisms, even by more than three logarithmic cycles. The use of the parameters of 60 MPa, 30 min, and 45 °C made it possible to obtain strawberry juice while maintaining good microbiological quality even for 12 weeks of storage at 6 °C [2]. SCCD also enables the inactivation of tissue enzymes such as polyphenol oxidase or peroxidase, thereby inhibiting the browning processes [2,3,5]. Penetration of a food sample with supercritical carbon dioxide causes a decrease in the pH inside the cell, physical disruption of the tissues, modification of cell membranes, and the extraction of intracellular compounds. In fruit and vegetable processing, SCCD is used primarily in liquid foods such as juices, where processing fruit caused significant tissue damage before preservation. Supercritical carbon dioxide is used in the food industry on a large scale for the extraction of caffeine from coffee beans, for the production of hop extracts, and for the de-alcoholization of wine. The use of SCCD has also been patented for, inter alia, the recovery of flavorings of seasonings and purification, fractionation and deodorization of oils. The fruit and vegetable preservation by SCCD technique has great potential, but is not yet used on an industrial scale [6].

The black currant contains 160–285 mg/100 g of ascorbic acid and 160–411 mg/100 g of anthocyanins, which are the most abundant representatives of polyphenols in that fruit [7]. Vitamin C and anthocyanins are well known for their anti-inflammatory, antibacterial, and neuroprotective properties. The high content of these antioxidants in blackcurrants makes this fruit a recommended dietary component in the prevention of cancer, cardiovascular diseases, and vision defects [8,9,10]. Vitamin C supports the production and preservation of collagen necessary for the formation of connective tissue. L-ascorbic acid facilitates the healing of wounds. It is an essential ingredient in the treatment of anemia thanks to increasing iron absorption and influencing the production of red blood cells. As a strong antioxidant, it participates in redox processes and is responsible for the destruction of free radicals [8,11]. The characteristic of blackcurrant anthocyanin profile consisting of the delphinidin and cyanidin rutinosides and glucosides, has application in detection adulteration in juices and wines. The intense dark red color, stability in acidic food, and the strong antioxidant properties of black currant make this fruit a great raw material for the production of juices and wines, as well as an additive in the production of functional food [11]. Studies in mouse models confirmed the possibility of anthocyanin extracts, obtained from Kenyan purple tea, crossing the blood–brain barrier. Due to their neuroprotective properties, they are promising dietary supplements for inhibiting oxidative stress in the brain and preventing neurodegenerative diseases such as Alzheimer’s, Parkinson’s, and multiple sclerosis [12].

There are indications that anthocyanins may have a positive effect on the body even though their absorption by the intestinal epithelium is very limited. The metabolism and absorption of these substances are complicated and not fully understood. However, to take full advantage of the health benefits that can be associated with the consumption of antioxidant-rich fruits, it is important to ensure the highest possible bioavailability and bioaccessibility of these compounds so that they can reach the target tissues and organs in the human body [10,13]. Bioaccessibility is defined as the amount of an active compound that is released from the matrix and is available for absorption into the bloodstream [14]. The bioaccessibility study was performed using an in vitro model that simulated digestion in the mouth, stomach, and small intestine along with dialysis through a cellulose membrane simulating the passive transport of components through the intestinal epithelium into the bloodstream.

**Figure 1:**
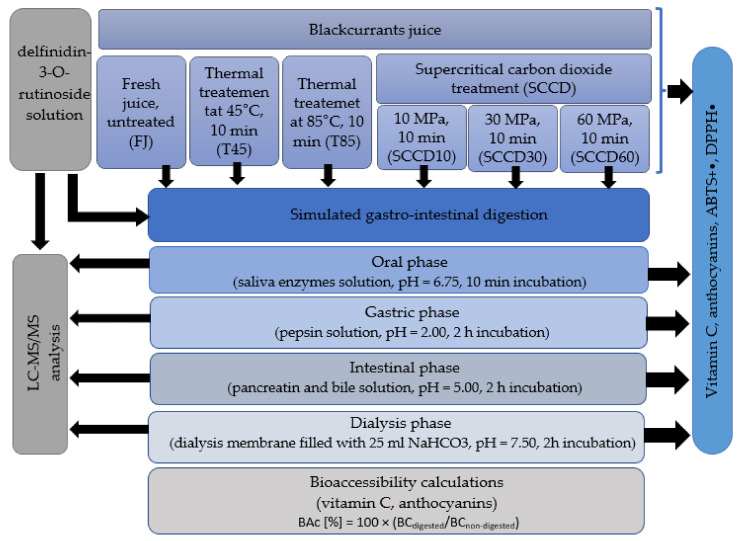
A schematic diagram showing the work flow described in this study.

Technological processing of food is one of the factors influencing the bioaccessibility of nutrients and bioactive compounds. The aim of the present research was to investigate whether SCCD also affects the stability and bioaccessibility of bioactive ingredients such as vitamin C and anthocyanins in blackcurrant juice after digestion in an in vitro gastrointestinal model. Moreover, the metabolites of selected anthocyanins after in vitro digestion and dialysis were detected using UPLC-PDA-MS/MS. The diagram of the work carried out in these studies is presented in Figure 1.

## 2. Results and Discussion

### 2.1. Stability and Bioaccessibility of Vitamin C after SCCD Treatment

According to the data presented in Table 1, the vitamin C content in the control samples of blackcurrant juice before digestion ranged from 150 mg/L in untreated juice to 172.54 mg/L in juice treated using SCCD at 60 MPa. SCCD improved the stability and extractability of the vitamin C from the tissue to the juice. Thermal treatment did not significantly affect the vitamin C content. The vitamin C content in blackcurrant juice was similar to the values obtained in other studies, which ranged from 60 to 280 mg/L depending on the country of origin of the fruit [11]. Marszałek et al. (2018) reported no effect of SCCD treatment under pressure of 10–60 MPa on the vitamin C content in apple juice [3]. The higher sensitivity of vitamin C was observed in strawberry juice, where about 30% of the vitamin C was degraded after SCCD treatment under a pressure of 30 and 60 MPa [1]. In the studies conducted on orange juice, SCCD caused a 12% reduction in vitamin C compared to the control samples, but a 43% decrease in this vitamin after pasteurization was also noted. Vitamin C is susceptible to degradation in the presence of oxygen and active oxidative enzymes. In turn, supercritical CO_2_ is a poor solvent for the extraction of this vitamin and does not contribute to its degradation. The replacement of the oxygen dissolved in the liquid juice matrix with supercritical CO_2_ could be beneficial for the better stability of vitamin C in products [15,16]. It transpires that the vitamin C contained in blackcurrant juice is resistant to the application of temperature. A low pH of the environment could have a protective effect on the chemical structure of vitamin C [17].

A total of 98% of vitamin C in the control samples was present in the l-ascorbic acid (AA) form. Due to digestion, it underwent reversible oxidation to l-dehydroascorbic acid (DHAA). During the simulated digestion in the small intestine, DHAA became the dominant form of vitamin C, reaching 76–93%. In the dialysate, the DHAA content again fell slightly, ranging from 29% in the FJ samples to 54% in the T45 samples. In the conditions prevailing in the digestive system, DHAA could also undergo further decomposition due to diketogulonic acid, 2-hydroxyfurfural, and other 5-carbon compounds [18].

The higher total vitamin C content in all treated and untreated samples were detected after first step of digestion in mouth in comparison to the same samples before digestion in mouth. Higher levels of vitamin C in samples after oral digestion compared to undigested samples may occur due to the activity of salivary enzymes. Enzymatic extraction of vitamin C from the juice tissue increased the content of this vitamin compared to non-digested samples. In contrast, the concentration of vitamin C significantly decreased after gastric digestion. Due to intestinal digestion and dialyse, the total vitamin C content decreased by 45–66% compared to the stomach stage. In our previous studies, we observed a decrease in the vitamin C content at the stage of oral digestion, and then, an increase in vitamin C concentration in the acidic environment of the stomach, which could have been caused by using a different matrix (puree). After intestinal digestion and dialysis, the vitamin C was significantly degraded (~98%). Studies conducted on blended fruit juice made from orange, pineapple, and kiwi showed high vitamin C stability after gastric digestion (recovery > 75%) and a significant decrease in vitamin C stability in an alkaline pH, which occurred in the intestinal step of digestion [17]. During the study of the bioaccessibility (BAc) of bioactive ingredients, including vitamin C from broccoli inflorescences, only a 7% loss of vitamin C after digestion in gastric conditions was noted, but as much as a 91% degradation in intestinal conditions [19]. Similarly, in the studies of pomegranate juice, a 95% loss of vitamin C after intestinal digestion was reported [18]. Vitamin C is commonly known to be an unstable bioactive compound sensitive to changes in pH (pH > 4), high temperature, enzyme activity, oxygen, light, and some metal ions [7].

It can also be observed that most of the vitamin C remaining after the simulation of intestinal digestion was passively transported across a cellulose membrane simulating the epithelial barrier of the small intestine. The most effective diffusion took place in samples treated using SCCD60 (87%), followed by the T45 sample (81%). Other processing conditions resulted in an absorption level of vitamin C ranging from 76 to 78%. The absorption of vitamin C in the human organism differs depending on the type of metabolite. Riboflavin and ascorbic acid are absorbed via a sodium-dependent active transporter (SVCT1) in the upper part of the small intestine. Dehydroascorbic acid can be absorbed thanks to glucose transporters and facilitated diffusion in the duodenum and jejunum. It is also worth mentioning that age, habits, medications taken, and physiological characteristics affect the high variability of the actual BAc of vitamin C ingested by the human alimentary tract [18,20].

**Figure 2:**
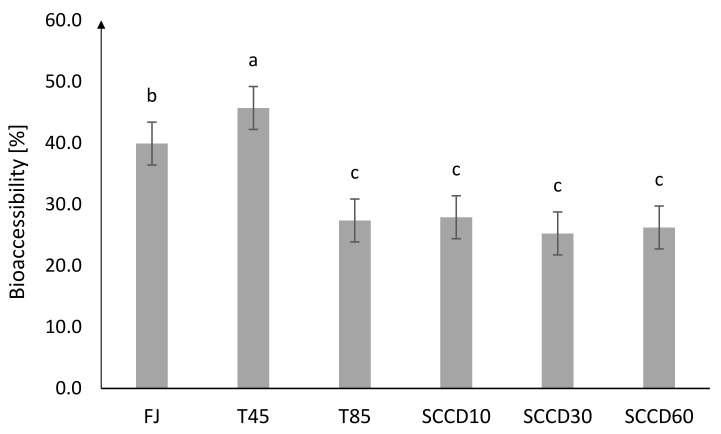
Bioaccessibility of total vitamin C in the dialysate after different processing: FJ-fresh juice; T45-thermal treated juice at 45 °C; T85-thermal treated juice at 85 °C; SCCD10-juice after supercritical carbon dioxide treatment at 10 MPa; SCCD30-juice after supercritical carbon dioxide treatment at 30 MPa; SCCD60-juice after supercritical carbon dioxide treatment at 60 MPa (The same letter above the bars indicates no significant difference between the mean (*p* ≤ 0.05) bioaccessibility of the samples after the different type of treatment).

The highest BAc of total vitamin C was noted for FJ (40%) and T45 (46%) (Figure 2). BAc after T85 and SCCD treatment was about 25–28%. SCCD did not improve the BAc of vitamin C. The effect of a lower pressure level in the SCCD treatment was insignificant compared to other pressure parameters and pasteurization. The initial higher vitamin C stability in the samples treated by SCCD compared to the thermal and control samples does not make the BAc of vitamin C higher in these samples. The digestive processes that took place in the simulated in vitro digestion model had a great influence on the Bac of vitamin C. To the best of our knowledge, there are no studies on the BAc of vitamin C in samples treated using SCCD. In our previous research, the influence of High Hydrostatic Pressure (HHP) on the BAc of antioxidants in blackcurrant puree was indicated. We reported that the BAc of vitamin C in dialysate was unfortunately very low and reached only 1% in samples treated using HHP in 400 and 600 MPa. Due to the possibility of the absorption of vitamin C just after gastric digestion, BAc after that stage was considered and ranged between 60% in pasteurized samples and 90% in HHP 400 and 600 MPa [7]. The differences in the bioaccessibility obtained in our studies could largely be due to using a different food matrix, which was also noted by other researchers. For instance, polyphenols contained in wine were more readily bioaccessible than those derived from grapes. In the case of solid food matrices, digestion at the mouth and stomach stages plays a very important role. The process of mastication in an appropriate pH and the presence of digestive enzymes, provides additional extraction of bioactive compounds and is essential for their release and availability for absorption [21].

In other studies, the BAc of vitamin C varied depending on the type and composition of the food matrix and the method of assessing the absorption of substances into the bloodstream (centrifugation or dialysis). Rodríguez-Roque et al. [17] who used a dialysis membrane in their studies, reported a BAc level of vitamin C from blended fruit juice of 15%. In other studies, also using a cellulose membrane, the BAc of vitamin C was obtained at a lower level of approx. 2.5% (in pomegranate juice) and 3.2% (in broccoli inflorescences) [18,19]. Among the studies using the centrifugation technique to assess BAc, Cilla et al. (2012) noted large differences in the BAc of vitamin C depending on the addition of a protein component to the mixed fruit juice and the processing technique. HHP treatment (400 MPa, 40 °C, 5 min) did not improve the BAc of vitamin C compared to short thermal treatment (90 °C, 30 s).

### 2.2. Stability and Bioaccessibility of Anthocyanins and Determination of Delphinidin Metabolites after In Vitro Gastro-Intestinal Digestion

The total anthocyanin content in blackcurrant juice in the control samples ranged from 1 173 mg/L in the SCCD30 samples to 498 mg/L in the T85 samples (Table 2). The SCCD treatment of the juice positively influenced the retention and extractability of anthocyanins, which, regardless of the pressure applied, were higher than in the fresh juice. The best effect was obtained using SCCD treatment at 30 and 60 MPa. On the other hand, pasteurization at 85 °C definitely contributed to the degradation of anthocyanins. Thermal treatment at 45 °C did not have a significant effect on the total anthocyanin content. Four major anthocyanins were determined in the blackcurrant juice, in descending order of concentration: delphinidin-3*-O-*rutinoside (df-3*-O-*rut), cyanidin-3*-O-*rutinoside (cy-3*-O-*rut), delphinidin-3*-O-*glucoside (df-3*-O-*glu), and cyanidin-3*-O-*glucoside (cy-3*-O-*glu). Other studies confirmed that SCCD treatment allows maintaining the high stability of anthocyanins derived from plants [6], thanks to the inactivation of the polyphenol oxidases and reduction in the activity of peroxidases. Processing using the SCCD method may result in the occurrence of reversible changes in the structure of proteins, such as enzymes, and inactivate them [22]. As a result of the SCCD treatment (30 and 60 MPa, 45 °C, 30 min) on strawberry juice, anthocyanin degradation following first-order kinetics was observed. The processing temperature and time were the most important for preservation conditions followed by pressure [1]. In the other studies, SCCD treatment at 23 MPa decreased the anthocyanin content compared to the control samples of blood orange juice by about 8%, which did not differ significantly from the values obtained for the thermal treated samples (88–91 °C, 30 s). On the other hand, the application of 13 MPa showed a slight loss of anthocyanins compared to the control samples, and the stability of the anthocyanins was higher than in the pasteurized samples [16].

As a result of digestion at the oral stage, the total anthocyanin content decreased in all samples. In the FJ and T45 samples, there was a decrease of 20–21%, in the T85 of 36%, and in the samples treated with SCCD by as much as 61–72%. After digestion in gastric conditions, a further decrease in the total anthocyanins in the FJ and T45 samples of 22% was recorded. In the remaining samples, the anthocyanin content increased in the range of 6% in SCCD60 to 43% in T85. The total anthocyanin content after simulated gastric digestion was the highest in the FJ samples. Insignificant differences were observed in the remaining samples. The digestion at the small intestine and after dialysis stage decreased the amount of total anthocyanins in all samples by 85–90%. Less anthocyanins were absorbed by a membrane into the dialysate compared to vitamin C. The amount of anthocyanins in the dialysate was higher only in the FJ samples than detected on the other side of the membrane. Finally, insignificant differences were observed between SCCD10 and the FJ sample after dialysis.

Other studies have confirmed a decrease in anthocyanins as a result of simulated digestion. The conditions reflecting those in the stomach were the most favorable for anthocyanin stability. On the other hand, at the small intestine digestion stage, there was a significant decrease in the anthocyanin content [7,21,23]. This phenomenon was explained mainly by changes in pH, which are one of the factors that significantly influence the structural changes of anthocyanins. An alkaline pH contributes to the aglycone ring breakage and formation of insoluble polymeric brown pigments [24]. In the studies conducted on blackcurrant puree, a more pronounced increase in the anthocyanin content was noticed during simulated gastric digestion. In a more complex matrix, containing more plant tissue and pectin than the juice, pepsin digestion and the effect of an acidic pH played a greater role in the extraction of bioactive compounds [7]. Anthocyanins are located in the vacuoles of cells, therefore, after consuming less processed fruit, the release of these compounds occurs during digestion [25].

The mechanisms of anthocyanin absorption in the gastrointestinal tract have not been thoroughly investigated. In vivo studies on humans and animals, based on the analysis of anthocyanins concentration in blood and tissues, provide evidence that, apart from the small intestine, they can be partially absorbed in the oral cavity [26], and also in a glycosidic form through the active transporters in the gastric mucosa [27]. The microbiota of the large intestine is also of great importance for the metabolism and absorption of anthocyanins [24,28].

**Figure 3:**
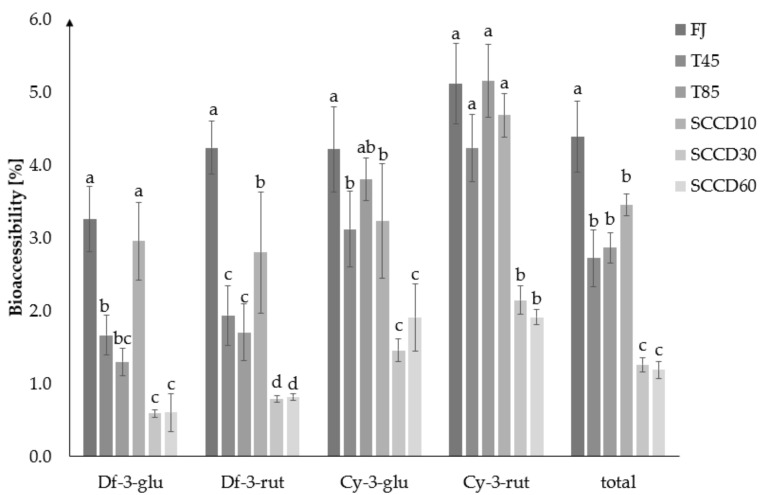
Bioaccessibility of blackcurrant juice anthocyanins in the dialysate after different processing: FJ-fresh juice; T45-thermal treated juice at 45 °C; T85-thermal treated juice at 85 °C; SCCD10-juice after super critical carbon dioxide at 10 MPa; SCCD30-juice after super critical carbon dioxide at 30 MPa; SCCD60-juice after super critical carbon dioxide at 60 MPa. (The same letter above the bars indicates no significant difference between the mean (*p* ≤ 0.05) bioaccessibility of the samples after the different type of treatment).

The BAc of total anthocyanins was the highest in fresh juice (4.4%), then in the T45, T80 and SCCD10 samples (2.7–3.4%), the lowest in SCCD30 and 60 (1.3 and 1.2%) (Figure 3). SCCD did not improve the BAc of total anthocyanins compared to the thermally treated and untreated samples. The most accessible glycoside was cy-3*-O-*rut, in all types of samples. Cyanidins turned out to be more bioaccessible than delphinidins. Among the SCCD variants, the highest BAc of total anthocyanins was obtained after the application of 10 MPa. The SCCD treatment at 10 MPa improved the BAc of df-3*-O-*glu and df-3*-O-*rut compared to the samples subjected to thermal treatment at 45 and 85 °C. Peixoto et al. (2018) reported in a study of anthocyanins derived from blueberries that delphinidin derivatives were degraded by simulation of digestion and were the least bioaccessible glycoside. The greater the number of –OCH_3_ groups in anthocyanin, the less susceptible the molecule was to convert or decay to undetectable forms. Consequently, these compounds were more available for absorption [29]. Delphinidin and malvidin derivatives in acetylated form maintained a high resistance to pH changes and lower degradation under digestive conditions. These can be one of the metabolites that are stable in the gastrointestinal tract and therefore enhance the antioxidant status of the blood plasma [24].

Irrespective of the structure of different anthocyanins, the metabolites released after enzymatic and/or acidic hydrolysis may play an important role in human health.

To better understand this phenomenon, UPLC-PDA-MS/MS analysis were used to determine anthocyanin metabolites during digestion in model conditions (Table 3). The results obtained showed that the major metabolite of df-3*-O-*rut, the most abundant anthocyanin in blackcurrants, was protocatechuic acid. This compound was present in an amount possible to quantify after the first step of digestion with the use of salivary enzymes, and after simulation of gastric digestion. It was not detected in the intestinal and dialyse stage, possibly due to the low concentration caused by sequential dilutions (Table 4). Depending on the material tested, different anthocyanin metabolites have been found in the available publications. Our results have been confirmed in some in vivo studies on humans [30,31]. Mallery et al. (2011) conducted clinical studies on the effect of oral digestive conditions on metabolism and bioactivation of black raspberry anthocyanins using the LC/MS-MS technique. An analysis of the salivary samples after rinsing the mouth with preparations based on lyophilized black raspberry showed the presence of the parent anthocyanins as well as protocatechuic acid as a functional, stable metabolite, up to 4 h after administration [30]. Other studies dealt with the in vivo metabolism of cyanidin glycosides in blood orange juice (HPLC/MS/MS). A total of 44% of the metabolites detected in the blood plasma collected within 24 h after juice consumption were protocatechuic acid, a significant amount was also detected in the fecal samples, but not in the urine. It was shown that after digestion and absorption into the body, 73% of the ingested cyanidin glycosides turned into protocatechuic acid. This metabolite may have contributed to the short-term increased antioxidant capacity of blood plasma after the consumption of cyanidin-rich foods [31]. Goszcz et al. (2017) found that delphinidin was a very unstable anthocyanin (t1/2~30 min) and the main product of its degradation was gallic acid. The degradation of delphinidin was investigated by LC-MS/MS analysis of solutions incubated for 30 min in phenol red-free tissue culture medium (pH 7.4, temperature 37 °C). It was shown that at a concentration of 100 µM of both delphinidins and gallic acid showed cytotoxicity to a cultured cell model of the human umbilical vein endothelium, and had a protective effect on the cells at lower concentrations (100 nM–1 µM) [32]. In the in vivo studies of the metabolism of berry anthocyanins in humans, it was found that during 48 h of observation after consuming bilberry-lingonberry purée with and without oat cereals, the phenolic acid content in urine increased. Homovanillic and vanillic acids have been detected as the main metabolites of anthocyanins. As in this study, gallic acid was not found to be a breakdown product of delphinidin glycosides [33]. Other authors reported that hippuric acid was the main metabolite of anthocyanins detected in urine and the tissues of rats on a wild blueberry-enriched diet [34]. Phenolic acids are compounds that present very good antioxidant properties. The metabolites and degradation products catalyzed by the microbiota may be responsible for the health benefits attributed to anthocyanins [28].

The bioavailability of anthocyanins in in vivo studies is reported to be very low, ranging from 1–2%, as they are often found only in slight amounts in urine, blood, and target organs. However, this value may be underestimated as the calculations do not consider all metabolites, activity of the microbiota in the anthocyanin’s degradation, and conjugates that may have an additional effect on the bioavailability of anthocyanins. The proven beneficial effects of consuming anthocyanin-rich foods on many health aspects further indicate the shortcomings of these data [10].

In our previous studies, the BAc of anthocyanins calculated as the ratio of the substance in the dialysate to that in the control samples was very low (maximum 1.6% in fruit crushed in a mortar). No improvement in the BAc of anthocyanins was observed using HHP treatment, irrespective of pressure conditions. However, due to the possibility of absorption of the glycoside form of anthocyanins from the stomach, BAc after this digestive step was also considered. The BAc of total anthocyanins after simulation of gastric digestion was significantly higher in the samples treated with HHP than with thermal pasteurization, reaching the highest value after the application of 600 MPa (68%) [7].

There are studies showing the beneficial effect of the SCCD technique on bioaccessibility when used in the pharmaceutical industry to complex the active substance of a drug onto polymeric carriers such as silica or cyclodextrin. SCCD activation and SCCD-assisted impregnation methods have proved to be promising strategies to obtain better quality medical preparations [35]. However, with regard to food-derived substances, only sparse research was found and all of it concerned the effect of SCCD on the BAc of lipophilic compounds such as carotenoids. Zhao et al. [36] investigated the effect of SCCD at a pressure of 10, 20, and 30 MPa, at 55 °C for 10, 20, 40, and 60 min on the physicochemical properties, isomerization, and in vitro BAc of lycopene in tomato juice. The effect of the SCCD parameters on phenolics was different, depending on the type of compound detected. The application of 10 MPa did not show a positive effect on the total phenolic compound content compared to the control samples, but it positively influenced the caffeic and chlorogenic acid content compared to the heat-treated samples. Conditions of 20 and 30 MPa had a positive effect and in particular the use of 30 MPa for 40 and 60 min had a more positive effect on the phenolic compound content (myricinic acid, ferulic acid, naringin, and chlorogenic acid) compared to the control. The BAc of lycopene increased significantly after treatment at 20 MPa for 20, 40, 60 min compared to the control and heat-treated samples. The SCCD samples at 20 MPa for 10 min, and 10 MPa at all pressure-holding times also showed a higher BAc of lycopene than the control and thermally treated samples. Damage to the cell barriers and sample homogenization may be the reason for the improvement in BAc. SCCD supports the release of bioactive ingredients from the food matrix [36]. It was found that SCCD increased the content of *cis*-lycopene isomers, which are more accessible than the *trans* isomers. Other researchers noticed that SCCD caused an increase in the volume of the proportion of small particles, while there was a decrease in the volume of the proportion of large particles in the settling pulp of orange juice compared to untreated juice, thus having a homogenizing effect on the food matrix. The cause of this process was the induction of high internal stresses causing the breakdown of particles during CO_2_ removal from the vessel [37,38]. In the research of Ubeyitogullari (2018), SCCD was used to produce low-crystallinity phytosterol nanoparticles by impregnating phytosterols into nonporous starch aerogels. The use of this technique allowed for a twenty-fold increase in the BAc of phytosterols [39]. In a study of the BAc of lycopene extracted using SCCD from tomato pomace, it was shown that it was about 2.4 times higher compared to hexane extraction. Extracts rich in cis-lycopene, obtained using SCCD treatment can be used to design novel functional foods while increasing the use of by-products of the tomato processing industry [40]. SCCD processing may affect the BAc of polyphenols by increasing the extractability of these compounds under pressure. Moreover, penetration of the sample by CO_2_ changes the pH of the environment to more acidic, which may contribute to the hydrolysis of polyphenols to simpler phenolic compounds [3].

### 2.3. Effect of Processing on the Antioxidant Capacity of Blackcurrant Juice in a Simulated Digestive System

**Figure 4:**
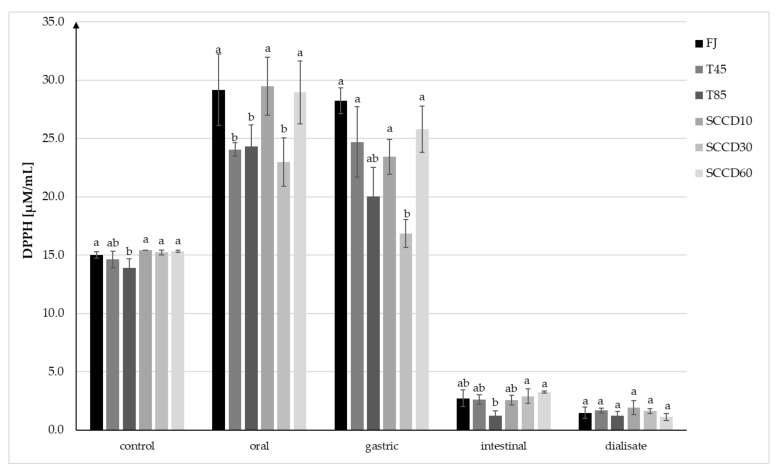
Antioxidant capacity (AC) of blackcurrant juice determined using the DPPH• assay at each step of digestion after different processing: FJ-fresh juice; T45-thermal treated juice at 45 °C; T85-thermal treated juice at 85 °C; SCCD10-juice after super critical carbon dioxide at 10 MPa; SCCD30-juice after super critical carbon dioxide at 30 MPa; SCCD60-juice after super critical carbon dioxide at 60 MPa. (The same letter above the bars indicates no significant difference between the mean (*p* ≤ 0.05) within each digestion stage).

The antioxidant capacity (AC) in control samples measured using the method with DPPH• radicals ranged from 13.90 to 15.35 µM/mL TEAC and was statistically significantly higher in FJ and SCCD treated samples compared to T85 (Figure 4). AC increased after digestion at the oral and stomach stage, and then significantly decreased after digestion in intestinal conditions and in dialysate. Following simulated oral digestion, the AC of FJ, SCCD at 10 and 60 MPa increased significantly, while SCCD at 30 MPa, T45, and T85 statistically had a significantly lower AC. After simulated gastric digestion, the AC of the FJ and T45 did not change significantly, but in the other samples the AC decreased in the range of from 11% in the SCCD-treated samples at 60 MPa to 27% in SCCD treated at 30 MPa. Due to simulated digestion in intestinal conditions and dialysate, a significant decrease in AC was noticed in all the samples. In the intestinal stage, samples treated using SCCD in all the pressure variants had similar level of AC as FJ and T45 samples and were significantly higher than T85 samples. However, no statistically significant differences were noted between the AC after dialysis.

**Figure 5:**
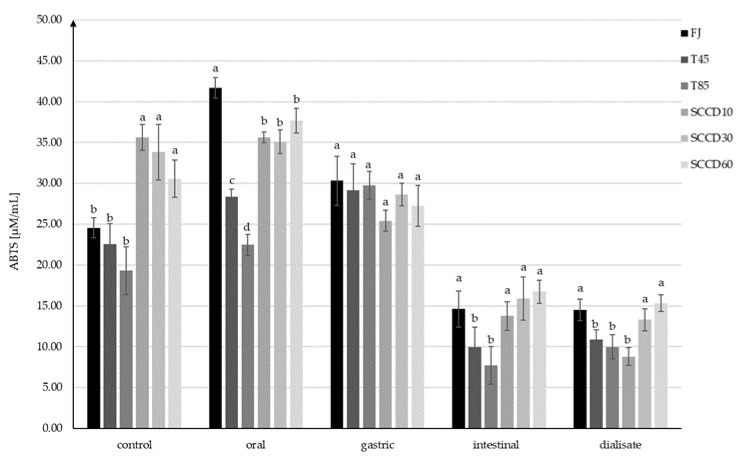
Antioxidant capacity (AC) using the ABTS+• assay at each step of digestion after different processing: FJ-fresh juice; T45-thermal treated juice at 45 °C; T85-thermal treated juice at 85 °C; SCCD10-juice after super critical carbon dioxide at 10 MPa; SCCD30-juice after super critical carbon dioxide at 30 MPa; SCCD60-juice after super critical carbon dioxide at 60 MPa. (The same letter above the bars indicates no significant difference between the mean (*p* ≤ 0.05) within each digestion stage).

Similar trends in the AC changes were confirmed by the results obtained using the ABTS+• assay, but the AC was significantly higher in all the SCCD-treated samples than in those subjected to thermal treatment and FJ samples (Figure 5). After digestion at the oral stage, there was a noticeable increase of AC in FJ, T45, T85, and SCCD60. The AC changes in SCCD10 and SCCD30 were insignificant. The highest AC after oral digestion was recorded in FJ samples, followed by SCCD, regardless of the type of pressure applied. T85 samples had the lowest AC. The increase in AC in FJ (70%) may have occurred due to the action of salivary enzymes, which contributed to the release of bioactive compounds from plant cells into the juice. Likewise, in other studies, at the earlier stages of digestion, a greater release of bioactive ingredients was noticeable in the non-processed samples. It was only at the intestinal stage that the advantage of the treated samples over the control ones in terms of antioxidant content could be observed [41]. Simulation of gastric digestion resulted in the unification of the AC to similar values in all the samples (25.4–30.3 µM/mL), with no statistically significant differences between them. Similarly to the DPPH• assay, there was a decrease in AC after the simulation of intestinal digestion; however, it was smaller than that observed using ABTS+• (ABTS+•: decrease of 39–74%; DPPH•: decrease of 83–94%). SCCD samples in all pressure variants were characterized by significantly higher AC than the samples subjected to thermal treatment and did not differ significantly from the FJ. This trend was maintained in the dialysate, with the exception of the SCCD10 sample, where the lowest AC was observed. The AC in the dialysate ranged from 8.8 to 15.3 µM/mL TEAC, and these values were similar to those at the intestinal stage. In contrast, thermal treatment at 45 °C did not cause an increase in AC, therefore the positive effect of SCCD treatment can only be attributed only to the application of supercritical carbon dioxide.

**Figure 6:**
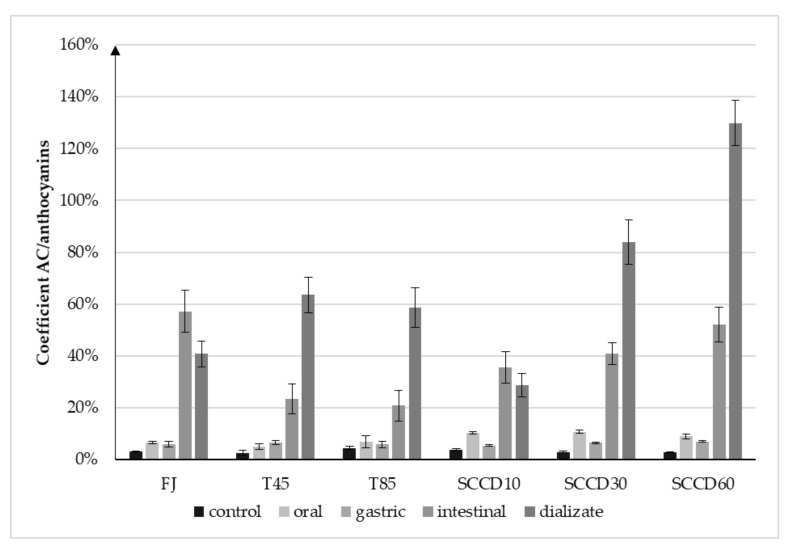
Coefficient of the ratio of increase the antioxidant capacity (AC) in relation to the total anthocyanins content in blackcurrant juice after different processing: FJ-fresh juice; T45-thermal treated juice at 45 °C; T85-thermal treated juice at 85 °C; SCCD10-juice after super critical carbon dioxide at 10 MPa; SCCD30-juice after super critical carbon dioxide at 30 MPa; SCCD60-juice after super critical carbon dioxide at 60 MPa.

The decrease in AC at each step of the digestive tract was significant, but much lower in comparison to the anthocyanins degradation. This phenomenon can be justified by the high AC of anthocyanins metabolites, whose concentration increased after digestion. The calculated coefficient expressed as antioxidant capacity per 1 mg/L of anthocyanins in blackcurrant juice (Figure 6) indicates that the AC increased significantly in relation to 1 mg of anthocyanins per liter. Considering the calculated coefficient in the dialysate step of digestion, the AC of the SCCD60 sample was three-fold higher, and the SCCD30 sample was two-fold higher than the control’s. This phenomenon may indicate the influence of SCCD on improvement the accessibility of antioxidants for digestion, thanks to which more metabolites were released and they could influence the AC of the fruit juice.

Other authors’ studies indicate the effectiveness of the SCCD method in maintaining the high antioxidant capacity of food products [42,43,44,45]. In the study of red grapefruit juice after SCCD treatment, at several pressure variants and times, no significant differences were detected in the total phenol content, ascorbic acid content, and AC compared to untreated juice [42]. Similarly, an examination of a hibiscus infusion treated using SCCD (34.5 MPa, 8% CO_2_, 6.5 min and 40 °C) indicated a slight loss of anthocyanins (9%) and no significant changes in phenolic compounds or AC during 14 days of storage [43]. The AC of apple juices subjected to SCCD at 35 °C, 15 min and pressure of 15 or 25 MPa turned out to be significantly higher with the use of higher pressure. Moreover, both pressures applied improved the AC compared to the fresh samples. As mentioned before, SCCD can promote conformational changes in the secondary structure of proteins and the inactivation of tissue enzymes [44]. The lychee juice treated with SCCD (8 MPa, 36 °C, 120 s) also showed higher AC measured using FRAP and ABTS+• assays, as well as more total phenols and flavonoids compared to thermal treated juice (100 °C, 60 s), and UHT (134 °C, 4 s). Treatment with SCCD supported preservation of the bioactive compounds due to the exclusion of oxygen and a mild temperature [45].

In our previous studies, AC measured using ABTS+• radicals also increased after oral digestion and additionally the DPPH• and ABTS+• assays were in line with the improvement in AC after gastric digestion and the lowering of AC in intestinal conditions [7]. The effect of simulated gastrointestinal digestion on the content of phenolic compounds and their antioxidant capacity in wild Chilean currants was investigated. As a result of digestion, there was an approx. 50% decrease in the content of total phenols and flavonoids, and approx. 80% in anthocyanins and hydroxycinnamic acids, as well as a decrease in antioxidant activity at each stage of digestion, correlated with the loss of bioactive compounds [46]. In studies conducted on strawberry juices enriched with inulin and oligofructose, treated with ultrasound, simulated gastrointestinal digestion resulted in a decrease in the content of phenolic compounds, flavonoids, and AC at each subsequent stage of the process. However, at the stage of the small intestine, higher AC (TEAC) and the content of the aforementioned bioactive compounds were noticed in the ultrasonically treated samples compared to the unprocessed ones [41].

**Figure 7:**
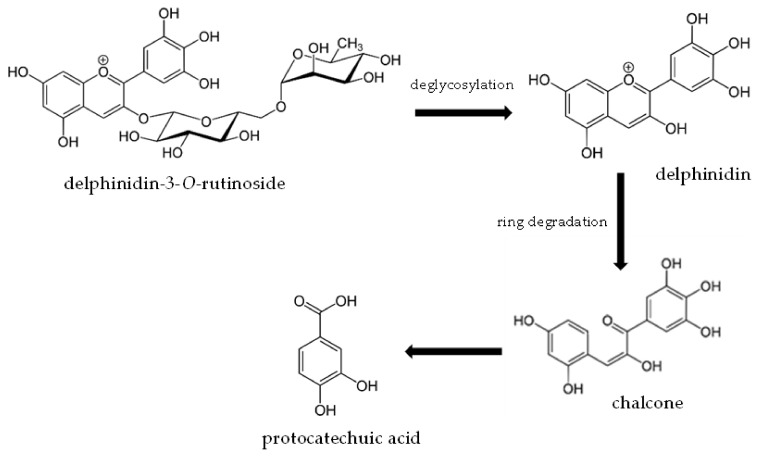
Proposed pathway for the formation of protocatechuic acid from delphinidin-3*-O-*rutinoside in the gastrointestinal tract.

The metabolites formed during the digestion of vitamin C and anthocyanins contribute remarkably to the antioxidant properties presented by the food products. For instance, protocatechuic acid detected in the samples in model conditions was the main metabolite of delphinidin-3*-O-*rutinoside. After the consumption of blood orange juice, as a source of anthocyanins, by six healthy subjects, high plasma concentrations of protocatechuic acid were noted, which contributed to an increase in short-term antioxidant activity [31]. Protocatechuic acid is one of the many metabolites that can be formed as a result of anthocyanin digestion in the gastrointestinal tract. The pathway of its formation from delphinidin-3*-O-*rutinoside is shown in Figure 7, according to available publications [6,47].

Bouayed et al. (2011) [23] showed that the AC (FRAP and ABTS+•) of antioxidants in apples after digestion with dialysis was about 50% lower than the values originally contained in the pre-digested sample. According to available studies, AC is correlated with the antioxidant content at a given stage of digestion. In addition, AC is influenced by environmental conditions such as pH, the interactions of antioxidants with other matrix components (iron and other mineral ions, dietary fiber, proteins), and the chemical structure of the compounds. The free radical scavenging activity of polyphenols depends on the number and position of the hydrogen donating hydroxyl groups on the aromatic rings of the molecules. Therefore, aglycones indicate a higher AC than their glycosides [23].

It has also been found that due to its low stability, delphinidin does not have a significant influence on AC, but its metabolites may be responsible for strong antioxidant properties. The parent compound is not necessary to obtain high AC. This explains the paradox of the low bioavailability of polyphenols, including anthocyanins, while exerting strong antioxidant effects and health benefits [32].

## 3. Materials and Methods

### 3.1. Reagents and Solvents

Sigma-Aldrich (St. Louis, MO, USA) was the supplier of the dialysis tubing cellulose membrane (avg. flat width 25 mm) and most of the enzymes and reagents, as follows: mucin from the porcine stomach—type II, α-amylase, heat-stable, (TDF-100A, 24,975 U/mL), pepsin from the porcine gastric mucosa (250 U/mg solid), pancreatin from the porcine pancreas (8 × USP specifications), porcine bile extract, sodium dodecyl sulfate—ACS reagent, sodium bicarbonate ≥ 99.5%, 2,2′-Azino-bis(3-ethylbenzothiazoline-6-sulfonic) acid, diammonium salt (ABTS+• radical), 2,2-diphenyl-1-picrylhydrazyl (DPPH• radical), (±)-6-hydroxy-2,5,7,8-tetramethyl-chromane-2-carboxylic acid (Trolox), dl-dithiothreitol (HPLC) (DTT), phosphoric acid 85%, acetonitrile (HPLC), formic acid ≥ 95.0%, and sodium hydroxide pellets ≥98.0%, (NaOH).

Other reagents were obtained from Chempur (Piekary Śląskie, Poland), such as: di-sodium hydrogen phosphate anhydrous pure p.a. ≥ 99.0% (Na_2_HPO_4_), di-potassium hydrogen phosphate (K_2_HPO_4_), sodium chloride pure p.a. ≥ 99.9% (NaCl) and di-sodium edetate standard solution 0.01 mol/L (EDTA). Hydrochloric acid pure p.a. ACS reagent 37% (HCl) and potassium peroxodisulfate ≥ 99.0% were purchased from Honeywell Fluka (Seelze, Germany). Ethanol—96% CZDA and methanol (HPLC grade) came from Avantor (Gliwice, Poland).

### 3.2. Testing Material

#### Preparation of Blackcurrant Juice

The research material was juice made from frozen blackcurrants (*Ribes nigrum* L.) of the *Tisel* cultivar, which is of Polish origin and bought from the local wholesaler. To produce blackcurrant juice, the fruit was enzymatically treated with a pectinolytic preparation (Klerzyme 150, DSM, Lille, France) for 1.5 h at 45 °C, and then squeezed in a hydraulic layer press (Tako, Czestochowa, Poland). The juice was transferred into 250 mL glass bottles and divided into six portions. The first was left without further processing (FJ). The second was heated to 45 °C for 10 min (T45), the third was pasteurized at 85 °C for 10 min (T85), both thermal processes were carried out in a laboratory pasteurizer (Labo Play, Bytom, Poland). The next portions were treated with supercritical carbon dioxide in the batch system using a Spe-ed SFE 4 (Applied Separations, Allentown, PA, USA). The method consists of placing the sample in a thermostatic pressure chamber, to pump CO_2_ at the applicable pressure, and then leave it for a specified period of time to ensure penetration. Three pressure variants were applied: 10, 30, and 60 MPa for 10 min, at 45 °C (SCCD10, SCCD30, SCCD60). Selected pressure parameters cover the entire operating range of the device, from minimum to maximum, with the optimal duration time of the process. Pasteurization carried out at 85 °C for 10 min is a widely used method of preserving fruit products in bath pasteurization. Mild heat treatment at 45 °C was used to exclude the influence of temperature in the treatment using SCCD. The samples were frozen before carrying out further research. Samples not subjected to digestion were considered to be control samples.

### 3.3. In Vitro Digestion Model with Dialysis and Calculation of Bioaccessibility

In vitro gastrointestinal digestion was conducted according to a slightly modified method presented by Buniowska et al. (2017) [48] and international consensus on the in vitro digestion method suitable for food [14]. Three replications of the juice samples, and distilled water as a blank sample, were poured into dark glass bottles and mixed with 5 mL of salivary enzyme solution (2.38 g of Na_2_HPO_4_, 0.19 g of K_2_HPO_4_, 8 g of NaCl, 100 mg of mucin and α-amylase with enzymatic activity 200 U/L, solution per 1 L of distilled water). The pH of the solution was then adjusted (HI 211 m, Hanna Instruments, Woonsocket, RI, USA) to 6.75 ± 0.20 by adding HCl (12 mol/L) or NaOH (2 mol/L). The solution was incubated in a shaking water bath (Labo Play, SWB 8N, Bytom, Poland) at 37 °C and 90 rpm for 10 min. Gastric digestion was then performed by adding pepsin solution and adjusting the pH to 2.0 with HCl (12 mol/L) and again incubating for 2 h. A total of 20 mL of each gastric digestion sample was placed in a clean bottle, titrated with NaOH (2 mol/L) to pH 5.00 ± 0.20, and 5 mL of pancreatin (1 g/L) and bile solution (25 g/L) were added to carry out the intestinal phase. The previously prepared dialysis membranes (cellulose, width 25 mm, length 30 cm) filled with 25 mL of NaHCO_3_ solution (0.5 M, pH 7.5) were immersed in the digested samples. The samples were incubated once more for 2 h under the same conditions. In order to finish the digestion simulation, the samples were cooled in an ice bath for 10 min. Samples were taken from each phase of the simulated digestion and frozen and stored until analysis. The dialysate solution inside the membrane is part of the sample potentially accessible to get into the bloodstream. Bioaccessibility (BAc) was determined using Equation (1) and is expressed as a percentage.

BAc [%] = 100 × (BC_digested_/BC_non-digested_) (1)

Equation (1). Calculation of bioaccessibility (Bac—bioaccessibility of bioactive compound; BC_digested_—the concentration of bioactive compound in the digested sample; BC_non-digested_—the concentration of bioactive compound in the non-digested sample).

### 3.4. Chemical Analysis

#### 3.4.1. Determination of Vitamin C

The vitamin C content was determined as a sum of l-ascorbic acid (AA) and l-dehydroascorbic acid (DHAA) according to the method introduced by Odriozola-Serrano et al. (2007) [49]. The sample was diluted using 0.01% phosphoric acid and filtered on a disposable syringe filter (0.45 µm, Macherey-Nagel, Düren, Germany). A solution of dithiothreitol (DTT) (1 g/L in 0.01% phosphoric acid) was used as the reducing agent to indicate the sum of AA and DHAA. Samples mixed with DTT in a 1:1 proportion were kept for 1 h in a dark place and at 4 °C before further analysis. The devices used for the analysis were: Waters chromatographic system (Milford, MA, USA), 2695 Separations Module, 2995 Photodiode Array Detector, Sunfire C 28 column, 5 µm, 4.6 mm × 250 mm with reversed phase and Sunfire C18 Sentry guard insert, 5 µm, 4.6 mm × 20 mm (both Waters, Milford, MA, USA). Samples were eluted isocratically using 0.01% phosphoric acid at a flow rate of 1 mL/min. Compounds were quantified using UV absorption at 245 nm. The amount of DHAA was calculated based on the difference between the sum of both acids and AA.

#### 3.4.2. Determination of Anthocyanins

The total anthocyanin content and their individual monomers were determined according to the method presented by Oszmiański (2002) [50]. The equipment and column used for the analysis were the same as described before in Section 2.3.2. The sample injection was 10 µL, and the analysis time was 26 min, the column was heated to 25 °C as before. A 4.5% aqueous formic acid solution (A) and an 80% acetonitrile solution in the previous formic acid solution (B) at a flow rate of 1.0 mL/min were the eluent. Anthocyanins were quantified using vis absorption at 520 nm. The amount of anthocyanins was calculated as cyanidin-3-glucoside.

#### 3.4.3. Antioxidant Capacity According to the ABTS+• Radical Assay

The method described by Re et al. (1999) [51] was the principle used to determine the antioxidant capacity with the ABTS+• radical. A total of 18 h before starting the analyses, a cationic radical solution was prepared by combining 7 mM ABTS+• and 2.45 mM potassium persulfate and was kept in a dark place. The radical solution was diluted with ethanol to an absorbance of 0.740–0.750 at a wavelength of 734 nm. The 1 mg/mL solution of Trolox in ethanol was the reference material, which was also used to prepare the standard curve. During the analysis, 0.025 mL of the sample, or distilled water as a blank, was dispensed into cuvettes, 2.5 mL of ABTS+• radical solution was added, mixed thoroughly, and incubated at 30 °C for 6 min. Absorbance was measured at 734 nm in ethanol. The antioxidant capacity was determined using Equation (2) and expressed as Trolox Equivalent Antioxidant Capacity (TEAC). The absorbance was measured with the use of a Pharmacia Biotech UV/Vis spectrophotometer, model Ultrospec 2000 (Amersham, UK).

AC [µM/mL] = ((A_0_ − A_s_) × 100 × a × D)/1000 (2)

Equation (2). Calculation of antioxidant capacity (AC) (A_0_—absorbance of the blank; A_s_—absorbance of the sample; a—coefficient from the standard curve (y = ax); D—dilution carried out during sample preparation and in the cuvette.

#### 3.4.4. Antioxidative Capacity According to DPPH• Radical Assay

The method presented by Yen and Chen (1995) [52] was the guideline used to determine the antioxidant capacity with the DPPH• radical. A solution of the DPPH• radical (1 mM) in methanol was prepared 3 h before starting the analyses and left to incubate in a dark place. After this time, it was diluted with 80% methanol to obtain a concentration of 0.1 mM and an absorbance in the range of 0.700–0.800. A standard curve was prepared using Trolox dissolved in methanol (1 mg/mL). A total of 2 mL of DPPH• solution and 0.1 mL of juice, or a blank sample, were mixed in a cuvette. After incubation at room temperature in the dark for 30 min, the absorbance of the samples was measured at 517 nm. A Pharmacia Biotech spectrophotometer (Ultrospec 2000; Amersham, UK) was also used this time. The antioxidant capacity was determined using Equation (2) and expressed as Trolox Equivalent Antioxidant Capacity (TEAC).

#### 3.4.5. Determination of Anthocyanin Metabolites in a Model System

##### Preparation of the Delphinidin-3-*O*-Rutinoside Solution

In order to recognize the metabolites that are formed after anthocyanin digestion, an aqueous solution of 0.05 mg/mL of delfinidin-3*-O-*rutinoside (Df 3*-O-*rut) was prepared and subjected to simulated digestion in the same conditions as the blackcurrant juice. The solution was subjected to LC-MS/MS analysis before digestion and after each digestion step.

##### LC-MS/MS Analysis

The analysis was carried out according to the methodology developed by Kapusta et al. (2018) [53]. Anthocyanin metabolites were investigated using a UPLC-PDA-MS/MS Waters ACQUITY system (Waters, Milford, MA, USA), with binary pump manager, sample manager, column manager, PDA detector, and tandem quadrupole mass spectrometer (TQD) with electrospray ionization (ESI). The separation was performed using a BEH C18 column (100 mm × 2.1 mm i.d., 1.7 µm, Waters) kept at 50°C. The applied solvents were: 2% formic acid in water *v/v* (mobile phase A) and 2% formic acid in 40% acetonitrile in water *v/v* (mobile phase B). The gradient program was set as follows: 0 min 5% B, 0–8 min linear to 100% B, 8–9.5 min for washing and return to initial conditions. Before injection, samples were filtered through a membrane filter (0.45 µm, Merck Millipore, Burlington, MA, USA) and injected directly into a chromatographic column. The injection volume was 5 µL (partial loop with needle overfill) and the flow rate was 0.35 mL/min. The parameters applied for TQD were: capillary voltage 3.5 kV; cone voltage 30 V in positive and negative mode; the source was maintained at 250 °C and the desolvation temperature was 350 °C; cone gas flow 100 L/h; and desolvation gas flow 800 L/h. Argon was used as the collision gas at a flow rate of 0.3 mL/min. The detection and identification of chemical compounds were based on characteristic PDA spectra, mass to charge ratio and ion fragments formed after collision-induced dissociation (CID). The quantitative analysis was possible only for one compound and was based on specific MS transitions in Multiple Reaction Monitoring (MRM) mode. The most intense transitions 611 > 303 were used for the quantification. Quantification was performed by injecting a standard solution of certain concentration ranging from 0.05 to 5 mg/mL. External standard calibration lines were generated by three repeated injections of standard solutions at seven concentration levels (0.05; 0.1; 0.25; 0.5; 1; 2.5; and 5 mg/L) at 1 day. A plot of peak area with respect to the corresponding concentration was used to demonstrate linearity. The linear regression equation and correlation coefficient were calculated by weighted (1/x^2^) least-squares linear regression analysis. Linearity was considered to be acceptable when correlation coefficients were R^2^ ≤ 0. Waters MassLynx software v.4.1 was used for data acquisition and processing.

### 3.5. Statistical Analysis

A statistical analysis of the results obtained was carried out using the Statistica 7.1 program (StatSoft, Tulsa, OK, USA). A one-way analysis of variance with the ANOVA test and a significance analysis of the differences in mean values were performed using Tukey’s test with a confidence level of α = 0.05. Each sample was digested in three independent trials and analyzed in duplicate.

## 4. Conclusions

Supercritical carbon dioxide (SCCD) processing at 10, 30, and 60 MPa for 10 min and 45 °C contributed to an improvement in the stability of vitamin C, total anthocyanins, and antioxidant capacity (AC) measured using the ABTS+• assay in blackcurrant juices. Thermal treatment at 85 °C was insignificant for the stability of vitamin C, but caused a significant degradation of total anthocyanins and a decrease in AC. As a result of simulated gastrointestinal digestion in the in vitro model, ascorbic acid was gradually oxidized to dehydroascorbic acid, and the total vitamin C and anthocyanin content decreased. No improvement in the BAc of vitamin C and total anthocyanins was reported after SCCD treatment. The positive effect of SCCD at 10 MPa was noted for the BAc of glycosides: delphinidin-3*-O-*glucoside and delphinidin-3*-O-*rutinoside compared to thermal-treated samples. Overall, cyanidins were more bioaccessible than delphinidins, led by cyanidin-3*-O-*rutinoside in all sample types. An inverse correlation between AC and BAc of antioxidants was noted. This may indicate the formation of metabolites that were impossible to detect, but showed a high antioxidant value. The calculated BAc concerns dialysate compounds in the parent form, while they may have degraded to undetectable forms that are still of high antioxidant value. The UPLC-PDA-MS/MS analysis of the digested delphinidin-3*-O-*rutinoside extract showed that the main metabolite of this glycoside is protocatechuic acid, distinguished by its high antioxidant activity. SCCD is a non-thermal processing technique that increases the extractivity of bioactive compounds and reduces their degradation by penetrating the sample using CO_2_ at supercritical state, while displacing oxygen, and inactivating tissue enzymes. The significantly higher antioxidant activity in the SCCD samples, especially at 30 and 60 MPa, than in the thermal samples, suggests that this technique may be an effective method of improving the health value of antioxidant-rich products.

## Figures and Tables

**Table 1:** l-ascorbic acid (AA), l-dehydroascorbic acid (DHAA) and total vitamin C content at each stage of the digestion of blackcurrant juice.

	Sample	AA (mg/100 mL)	DHAA (mg/100 mL)	Total Vitamin C (AA+DHAA) (mg/100 mL)
**control**	FJ	147.71 ^c^ ± 11.04	2.29 ^c^ ± 0.71	150.00 ^c^ ± 11.08
	T45	155.09 ^bc^ ± 3.56	3.17 ^ab^ ± 0.91	158.25 ^bc^ ± 3.14
	T85	155.81 ^bc^ ± 2.82	1.95 ^c^ ± 0.65	157.76 ^bc^ ± 2.60
	SCCD10	162.68 ^ab^ ± 8.80	3.23 ^ab^ ± 1.21	165.91 ^ab^ ± 9.36
	SCCD30	156.93 ^bc^ ± 5.60	3.84 ^a^ ± 0.38	160.77 ^bc^ ± 5.82
	SCCD60	169.39 ^a^ ± 2.66	3.15 ^ab^ ± 0.92	172.54 ^a^ ± 2.25
**oral**	FJ	188.72 ^bc^ ± 15.05	9.10 ^a^ ± 1.64	197.82 ^ab^ ± 16.59
	T45	201.55 ^ab^ ± 11.03	1.83 ^c^ ± 0.49	203.38 ^ab^ ± 11.44
	T85	180.99 ^c^ ± 12.66	6.96 ^b^ ± 1.56	187.95 ^b^ ± 13.71
	SCCD10	210.91 ^a^ ± 4.06	2.35 ^c^ ± 0.83	213.26 ^a^ ± 3.66
	SCCD30	201.71 ^ab^ ± 4.87	8.69 ^ab^ ± 1.26	210.39 ^a^ ± 4.95
	SCCD60	196.97 ^abc^ ± 3.59	7.93 ^ab^ ± 0.48	204.90 ^ab^ ± 3.12
**gastric**	FJ	170.03 ^a^ ± 14.71	2.73 ^c^ ± 0.51	172.77 ^a^ ± 14.42
	T45	161.06 ^ab^ ± 3.57	2.40 ^c^ ± 0.77	163.46 ^ab^ ± 3.73
	T85	151.76 ^b^ ± 9.45	8.19 ^ab^ ± 5.27	159.96 ^ab^ ± 9.56
	SCCD10	151.88 ^b^ ± 11.08	11.65 ^a^ ± 2.31	163.53 ^ab^ ± 9.43
	SCCD30	131.45 ^c^ ± 12.82	4.08 ^bc^ ± 1.09	135.52 ^c^ ± 12.79
	SCCD60	152.94 ^ab^ ± 5.37	1.87 ^c^ ± 0.62	154.81 ^b^ ± 5.49
**intestinal**	FJ	4.25 ^a^ ± 0.93	13.83 ^ab^ ± 2.18	18.08 ^a^ ± 2.93
	T45	2.19 ^bc^ ± 0.70	14.81 ^a^ ± 3.15	16.99 ^ab^ ± 3.67
	T85	1.50 ^cd^ ± 0.17	12.20 ^ab^ ± 2.45	13.70 ^bc^ ± 2.61
	SCCD10	2.53 ^b^ ± 0.36	10.79 ^b^ ± 1.72	13.32 ^bc^ ± 1.36
	SCCD30	1.06 ^de^ ± 0.36	10.94 ^b^ ± 1.12	12.00 ^c^ ± 0.72
	SCCD60	0.46 ^e^ ± 0.06	6.28 ^c^ ± 0.67	6.74 ^d^ ± 0.67
**dialysate**	FJ	42.61 ^a^ ± 4.33	17.26 ^b^ ± 1.69	59.87 ^b^ ± 5.87
	T45	33.61 ^b^ ± 2.50	38.75 ^a^ ± 5.30	72.37 ^a^ ± 3.09
	T85	23.04 ^c^ ± 1.21	20.17 ^c^ ± 3.36	43.21 ^c^ ± 4.14
	SCCD10	30.22 ^b^ ± 2.89	16.04 ^c^ ± 1.21	46.26 ^c^ ± 3.79
	SCCD30	21.94 ^c^ ± 3.47	18.69 ^c^ ± 2.21	40.63 ^c^ ± 1.84
	SCCD60	21.43 ^c^ ± 1.79	23.86 ^c^ ± 3.59	45.28 ^c^ ± 2.52

FJ-fresh juice; T45-thermal treated juice at 45 °C; T85-thermal treated juice at 85 °C; SCCD10-juice after supercritical carbon dioxide treatment at 10 MPa; SCCD30-juice after supercritical carbon dioxide treatment at 30 MPa; SCCD60-juice after supercritical carbon dioxide treatment at 60 MPa. The same superscripts letter indicate no significant difference between mean (*p* ≤ 0.05) in the columns within each digestion stage.

**Table 2:** Total anthocyanin content at each stage of digestion of blackcurrant juice.

	Sample	Df-3*-O-*glu (mg/L)	Df-3*-O-*rut (mg/L)	Cy-3*-O-*glu (mg/L)	Cy-3*-O-*rut (mg/L)	Total Anthocyanins (mg/L)
control	FJ	111.13 ^c^ ± 5.85	371.91 ^d^ ± 18.76	45.75 ^c^ ± 2.18	259.66 ^c^ ± 12.28	788.45 ^c^ ± 39.07
	T45	101.36 ^c^ ± 9.04	342.26 ^d^ ± 30.38	41.45 ^c^ ± 3.54	236.86 ^c^ ± 20.05	721.93 ^c^ ± 62.99
	T85	73.62 ^d^ ± 4.14	236.99 ^e^ ± 2.42	28.53 ^d^ ± 1.94	159.28 ^d^ ± 2.95	498.43 ^d^ ± 11.27
	SCCD10	138.80 ^b^ ± 19.38	460.60 ^c^ ± 31.44	56.08 ^b^ ± 7.35	306.72 ^b^ ± 27.17	962.20 ^b^ ± 25.31
	SCCD30	159.95 ^a^ ± 8.23	579.44 ^a^ ± 34.45	71.55 ^a^ ± 6.19	383.67 ^a^ ± 28.74	1173.02 ^a^ ± 64.69
	SCCD60	159.90 ^a^ ± 4.85	521.73 ^b^ ± 11.37	62.77 ^b^ ± 1.90	334.94 ^b^ ± 7.45	1079.34 ^a^ ± 25.30
oral	FJ	96.14 ^a^ ± 11.09	314.95 ^a^ ± 19.52	34.95 ^a^ ± 6.09	186.23 ^a^ ± 10.10	632.28 ^a^ ± 45.36
	T45	85.24 ^a^ ± 21.99	289.64 ^a^ ± 27.35	31.98 ^a^ ± 2.91	162.13 ^ab^ ± 18.40	568.99 ^a^ ± 59.44
	T85	44.97 ^b^ ± 13.15	153.49 ^b^ ± 16.45	17.25 ^b^ ± 5.37	105.52 ^c^ ± 22.08	321.23 ^b^ ± 27.05
	SCCD10	48.74 ^b^ ± 2.42	165.39 ^b^ ± 6.50	18.55 ^b^ ± 0.65	111.90 ^c^ ± 4.34	344.58 ^b^ ± 13.21
	SCCD30	49.97 ^b^ ± 8.19	150.17 ^b^ ± 2.04	19.05 ^b^ ± 3.26	108.77 ^c^ ± 10.14	327.95 ^b^ ± 20.68
	SCCD60	56.08 ^b^ ± 7.80	208.56 ^b^ ± 13.46	21.12 ^b^ ± 2.95	132.90 ^bc^ ± 9.04	418.66 ^b^ ± 21.29
gastric	FJ	66.37 ^a^ ± 6.28	237.17 ^a^ ± 15.89	27.14 ^a^ ± 2.56	165.86 ^a^ ± 6.94	496.55 ^a^ ± 26.55
	T45	54.24 ^c^ ± 6.95	238.86 ^a^ ± 13.54	22.11 ^b^ ± 2.31	126.41 ^c^ ± 16.47	441.62 ^b^ ± 26.36
	T85	60.33 ^ab^ ± 6.08	212.41 ^b^ ± 10.14	21.86 ^b^ ± 3.69	165.46 ^a^ ± 11.30	460.05 ^b^ ± 19.41
	SCCD10	66.57 ^a^ ± 1.54	228.10 ^ab^ ± 4.48	25.84 ^a^ ± 0.56	154.12 ^ab^ ± 3.29	474.62 ^ab^ ± 9.26
	SCCD30	62.22 ^ab^ ± 1.45	213.37 ^b^ ± 4.27	24.00 ^ab^ ± 0.38	143.22 ^b^ ± 2.57	442.82 ^b^ ± 8.53
	SCCD60	62.56 ^ab^ ± 2.98	212.83 ^b^ ± 7.91	23.97 ^ab^ ± 0.82	142.92 ^b^ ± 5.06	442.27 ^b^ ± 16.64
intestinal	FJ	1.88 ^d^ ± 0.73	11.11 ^c^ ± 1.88	1.03 ^b^ ± 0.31	11.53 ^b^ ± 1.62	25.55 ^c^ ± 2.30
	T45	4.13 ^a^ ± 0.49	18.59 ^a^ ± 1.83	2.27 ^a^ ± 0.69	17.40 ^a^ ± 2.55	42.39 ^a^ ± 5.28
	T85	3.17 ^bc^ ± 0.31	16.11 ^ab^ ± 2.67	1.91 ^a^ ± 0.29	15.86 ^a^ ± 2.33	37.04 ^ab^ ± 3.52
	SCCD10	3.40 ^abc^ ± 0.33	16.34 ^ab^ ± 1.16	2.05 ^a^ ± 0.10	16.91 ^a^ ± 0.62	38.69 ^ab^ ± 2.04
	SCCD30	3.77 ^ab^ ± 0.32	16.26 ^ab^ ± 3.04	2.03 ^a^ ± 0.26	16.78 ^a^ ± 1.80	38.83 ^ab^ ± 4.06
	SCCD60	2.80 ^c^ ± 0.64	12.88 ^bc^ ± 2.64	1.77 ^a^ ± 0.19	14.61 ^ab^ ± 1.27	32.05 ^bc^ ± 4.73
dialysate	FJ	3.63 ^a^ ± 0.79	15.72 ^a^ ± 0.95	1.93 ^a^ ± 0.29	13.29 ^a^ ± 2.11	34.56 ^a^ ± 3.94
	T45	1.67 ^b^ ± 0.14	6.53 ^c^ ± 0.91	1.28 ^b^ ± 0.14	9.98 ^b^ ± 0.90	19.46 ^b^ ± 1.63
	T85	0.96 ^b^ ± 0.16	4.03 ^d^ ± 0.93	1.08 ^b^ ± 0.08	8.21 ^bc^ ± 1.15	14.28 ^c^ ± 1.21
	SCCD10	3.96 ^a^ ± 0.83	12.51 ^b^ ± 2.27	1.76 ^a^ ± 0.22	13.96 ^a^ ± 2.59	32.19 ^a^ ± 5.41
	SCCD30	0.94 ^b^ ± 0.06	4.59 ^cd^ ± 0.25	1.04 ^b^ ± 0.08	8.17 ^bc^ ± 0.40	14.75 ^bc^ ± 0.73
	SCCD60	0.96 ^b^ ± 0.39	4.26 ^d^ ± 0.18	1.19 ^b^ ± 0.26	6.40 ^c^ ± 0.27	12.81 ^c^ ± 1.04

FJ-fresh juice; T45-thermal treated juice at 45 °C; T85-thermal treated juice at 85 °C; SCCD10-juice after supercritical carbon dioxide treatment at 10 MPa; SCCD30-juice after supercritical carbon dioxide treatment at 30 MPa; SCCD60-juice after supercritical carbon dioxide treatment at 60 MPa; df-3-*O*-glu-delphinidin-3-*O*-glucoside; df-3-*O*-rut-delphinidin-3-*O*-rutinoside; cy-3-*O*-glu-cyanidin-3-*O*-glucoside; cy-3-*O*-rut-cyanidin-3-*O*-rutinoside. The same superscripts letter indicates no significant difference between the mean (*p* ≤ 0.05) in the columns within each digestion stage.

**Table 3:** UPLC-PDA-MS/MS properties of delphinidin 3*-O-*rutinoside and protocatechuic acid as its major detectable metabolite (condition as in p. 3.4.5.2.).

No.	Compound	RT	[M − H]	Fragment Ions	Absorbance Maxima
		(Min.)	(*m*/*z*)	(*m*/*z*)	(nm)
1	Delphinidin 3*-O-*rutinoside	2.71	611^+^	303	277, 525
2	3,4-Dihydroxybenzoic acid (protocatechuic acid)	2.14	153^−^	109	260, 294

**Table 4:** The content of delphinidin-3-*O*-rutinoside (Df-3*-O-*rut) and protocatechuic acid at individual stages of the digestion simulation.

Stage of Digestion	Df-3*-O-*rut (µg/mL)	Protocatechuic Acid (µg/mL)
control	49.74 ± 0.89	0.0
oral	16.25 ± 1.34	0.63 ± 0.02
gastric	52.66 ± 3.25	0.44 ± 0.02
intestinal	3.65 ± 0.02	0.0
dialysate	0.0	0.0

## Data Availability

The original data presented in the study are included in the article, further inquiries can be directed to the corresponding author.

## Sample Availability

Samples of the compounds are not available from the authors.
